# Factors Associated within 28 Days In-Hospital Mortality of Patients with Acute Respiratory Distress Syndrome

**DOI:** 10.1155/2013/564547

**Published:** 2013-06-26

**Authors:** Nadia Sharif, Muhammad Irfan, Javaid Hussain, Javaid Khan

**Affiliations:** Pulmonary and Critical Care Section, Department of Medicine, Aga Khan University Hospital, Stadium Road, Karachi 74800, Pakistan

## Abstract

*Objective*. To determine the factors leading to in-hospital mortality within 28 days in hospitalized patients with ARDS. It was a prospective observational cohort study conducted in Intensive Care Unit of Aga Khan University Hospital Karachi from March to August 2011. *Methodology*. Data was collected from patients admitted in the intensive care unit on the basis of inclusion and exclusion criteria. The patients were followed daily for 28 days to record any in-hospital complications and the outcome of patients. *Results*. Total of 46 patients were included during this period out of which 56% (26) were males and 43% (20) were females. Mean age was 44 ± 19 years. There were 11 (23.9%) patients with age >65 and 35 (76%) had age <65 years. There were 21 (45.6%) patients with pulmonary ARDS and 25 (54.3%) had extrapulmonary ARDS. APACHE II score of >20 was present in 23 (50%) patients while the rest had score of <20. Regarding in-hospital complications, 23 (50%) patients developed sepsis, 31 (67.4%) had multiorgan failure, 14 (30%) had refractory shock, and 15 (32.6%) developed refractory hypoxemia. Out of 46 patients, 26 (56.5%) died within 28 days. On univariate analysis, high APACHE score, multiorgan failure, refractory shock, and refractory hypoxemia were main causes of death. *Conclusion*. ARDS is a syndrome of high mortality with mortality rate of 56.5% in this study. High APACHE, sepsis, multiorgan failure, refractory shock, and refractory hypoxemia are the leading causes of death in our patients.

## 1. Introduction

Acute respiratory distress syndrome (ARDS) is a form of severe hypoxemic respiratory failure characterized by bilateral lung infiltrates with no clinical or objective evidence of left heart failure [1]. This syndrome is among the major causes of morbidity and mortality in intensive care units (ICUS) worldwide. Even with optimal conventional therapies, the reported mortality rates in various studies vary from 30% to 70% [2, 3]. It can be caused by either direct or indirect insult to lung, hence, has been classified as pulmonary ARDS and extrapulmonary ARDS, respectively. This division is thought to have significant clinical implications as patients might respond differently to various treatments including mechanical ventilation [4, 5]. However, further studies [6] have failed to show any reproducible difference. Many studies have sought to identify factors during the acute illness that predict mortality [6–8]. These factors can be categorized as patient-, disease-, or treatment-related ones. Regarding patient-related factors older patients (age >65 yrs) and male gender (66%) are associated with increased death rates with ARDS [8–10]. Disease-related factors predicting mortality in various studies are underlying cause of ARDS [11], Acute Physiology and Chronic Health Evaluation (APACHE) [12], and in-hospital complications like sepsis [8], multiorgan failure (MOF), refractory shock, and refractory hypoxemia [13]. Among treatment-related factors protective ventilatory strategies have been shown to improve outcome in patients with ARDS [14]. 

Although ARDS is well studied worldwide, but no local data is available to document the factors associated with mortality in ARDS and difference in outcome in patients with pulmonary and extra pulmonary ARDS. Early identification of these factors will help to assess prognosis and aid in timely management. So the aim of this study is to evaluate the factors associated with mortality in our patients presented with ARDS.

## 2. Methodology

This was a prospective observational cohort study conducted in ICU of Agha Khan University hospital Karachi from March to August 2011. Study subjects include all adult patients (age greater than 14) with admitting diagnosis of ARDS. The patients with history or clinical evidence of congestive cardiac failure, patients with a do-not resuscitate order at ICU admission, or who died within 24 hrs of ARDS diagnosis were excluded from the study. *ARDS* was defined according to American-European Consensus Conference criteria (1): acute onset, PaO_2_/FiO_2_ of ≤200, and bilateral infiltrates on chest radiograph. Exclusion criteria were an occlusion pressure of ≥18 mmHg or clinical heart failure. Etiology of ARDS was broadly classified into *pulmonary ARDS* (which include any of these: pneumonia, aspiration pneumonia, inhalation injury, or lung contusions) and *extra pulmonary ARDS* (which include any of these: sepsis, pancreatitis, postblood transfusions, major trauma, or adverse reaction of drug). We prospectively identified variables that could be independently associated with mortality: age, gender, etiology of ARDS, high acute physiology and chronic health evaluation II (APACHE II) score, sepsis, multiorgan failure (MOF), refractory shock, and refractory hypoxemia. We broadly divide age into groups, that is, age >65 and age <65. APACHE score was also divided into two groups, that is, >20 and <20. *Sepsis* [15] was clinically recognized by the presence of two or more of the following: temperature >38.5°C or <35°C, heart rate >90 beats/min, respiratory rate >20 breaths/min or PaCO_2_ < 32 mmHg, WBC > 12,000 cells/mm^3^, <4000 cells/mm^3^ *and* are due to infection (culture or Gram stain of blood, sputum, urine, or normally sterile body fluid positive for pathogenic microorganism). *Multiorgan failure* [25] is defined to have presence of any of these in addition to ARDS—(1) acute renal failure: within 48 hours absolute increase in the serum creatinine concentration of ≥0.3 mg/dL from baseline, or oliguria of less than 0.5 mL/kg per hour for more than six hours, (2) cardiac arrest: reversible ventricular fibrillation or asystole, (3) CNS involvement: neuroimaging proven ischemic or hemorrhagic stroke or Glasgow coma scale <8 for ≥3 days, and (4) acute hepatic failure: bilirubin >5.0 mg/dL and prothrombin time or partial thromboplastin time >1.5 times control. Refractory shock was defined as need for dopamine at >15 mcg/kg/min, or norepinephrine or epinephrine at >0.25 mcg/kg/min to maintain mean BP at >60 mmHg. Refractory hypoxemia was defined as being unable to maintain PaO_2_ > 60 mmHg despite being on maximum ventilator support. The outcome measure was in-hospital mortality within 28 days.

Data was collected from patients admitted in the intensive care unit on the basis of inclusion and exclusion criteria. The purpose of the study was explained to the patients or next of kin of the patients and informed consent was taken. The age, gender, etiology, and APACHE score on day 1 were noted. The patients were followed daily for 28 days to record any in-hospital complications leading finally to in-hospital mortality.

## 3. Statistical Analysis

 Data was analyzed by using SPSS (Statistical Package for Social Sciences) version 19.0. A descriptive analysis was done for qualitative variables, that is, pulmonary ARDS, extra pulmonary ARDS, age (<65 or >65), APACHE score (<20 or >20), presence or absence of sepsis, multiorgan failure, refractory shock, and refractory hypoxemia, and was presented as frequency (percentage). Frequency of outcome variable, that is, mortality, was calculated and cross-tabulation was done between outcome variable and different factors. Differences between survivors and nonsurvivors were explored with chi-square test. A *P* value of <0.05 was considered significant.

## 4. Results

A total of 46 patients were included in this study on the basis of inclusion and exclusion criteria during 6-month period. Mean age was 44 ± 19 years. Out of 46 patients 11 (23.9%) had age greater than 65 years and 35 (76%) had age less than 65. There was slight male dominance in this cohort with 26 (56.5%) were males and 20 (43.5%) were females. Heart failure was excluded clinically and none of the patients underwent pulmonary artery catheterization.

**Figure 1:**
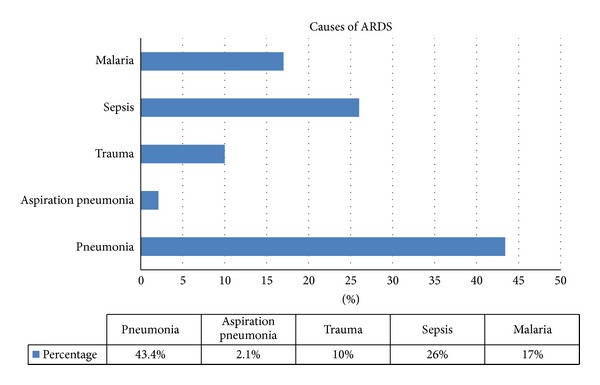
Frequency of causes of ARDS.

The causes of ARDS are shown in Figure 1. The predominant etiology was extra pulmonary ARDS, that is, 25 (54.3%). Interestingly 23 (50%) of patients had APACHE score of >20 and the rest had score of less than 20. Regarding in-hospital complications multiorgan failure was the predominant one 31 (67.4%) followed by sepsis 23 (50%), refractory hypoxemia 15 (32.6%), and refractory shock 14 (30%).

**Figure 2:**
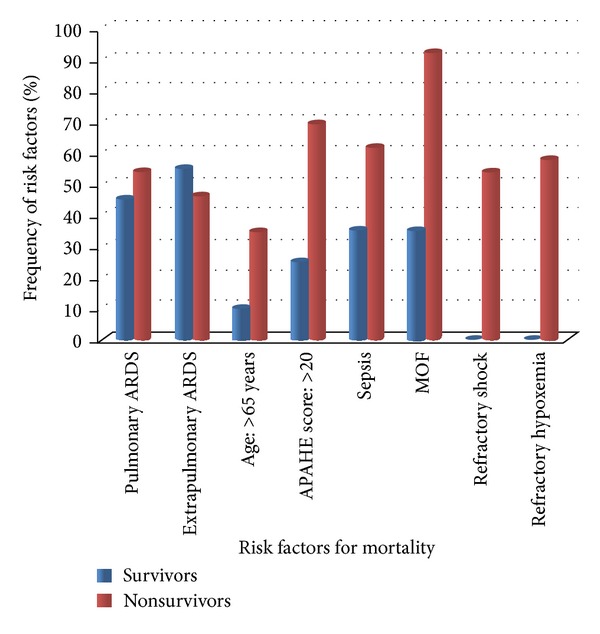
Comparison of risk factors among survivors and non survivors.

The primary outcome measure, that is, mortality, was 56.5% (*n* = 26). Mortality was higher in patients with pulmonary ARDS but did not reach the statistical significance. Out of 26 patients who died 14 (53.8%) patients had pulmonary ARDS while 12 (46.2%) patients belonged to extra pulmonary group. Comparison of risk factors of among survivors and non survivors is presented in Figure 2.

There was no significant difference between survivors and non survivors in sex distribution and age. Similarly sepsis was common among non survivors but did not reach the statistical significance 16 (61.5%, *P* value 0.07). Factors significantly associated with mortality (Table 1) were APACHE score of >20 (*P* = 0.003), multi organ failure (*P* ≤ 0.001), refractory shock (*P* ≤ 0.001), and refractory hypoxemia (*P* ≤ 0.001).

## 5. Discussion

This was a small study done in a single tertiary care center in Karachi. In our study of 46 patients, the mortality rate was 56%. In past two decades there are studies from world best centers claiming that mortality has decreased to up to 30% [16, 17], which may have been a result of improvement in the specific management of patients with ARDS as well as in the general management of ICU patients. But in this same era of lung protective ventilatory strategy, there are other studies [2, 18] still reporting mortality rate of 58%. In recent years there is increasing interest worldwide to find the individual predictors of mortality in ARDS.

A large number of these studies have been performed in Caucasians. Only a few studies are available from Asian countries including India. In Pakistan, to our knowledge, so far no study has been published on ARDS and mortality secondary to it. In consideration of this marked heterogeneity among studies, making a comparison of results from this study with the previously published literature is a challenging task.

The age distribution of patients among studies is variable. In some studies the majority of patients were younger, below 60 years of age [6], while others comprise elderly patients in majority [9, 19]. In our study 76% of the patients were below 65 years. This is maybe due to the fact that with such limited resources, younger population has better access to treatment facilities as compared to older population who are treated conservatively rather than shifting to ICU. Although patients with age above 65 years accounted for only 24% of study subjects (11/46), there was a suggestion of increased mortality compared with those below 65 years: 81.8% (9/11) versus 48.5% (17/35), respectively. So our results are consistent with previous studies, suggesting old age as risk factor for mortality in ARDS.

Male gender is associated with higher mortality rate with ARDS as assessed by International Classification of Diseases, Ninth Revision codes on death certificates [20]. This may be because of genetic factors that predispose to ARDS, because of differences in susceptibility to risk factors for ARDS or hormonal difference. Moss and Mannino have shown in their study that mortality rate is higher in male patients as compared to females [20]. They attribute it to genetic difference or response to the therapy. In a study by Seeley et al. [21], mortality rates were higher among females 51% versus 36%. However, in a study in India the mortality rates were again higher among men, that is, 57% [6]. However, in our study there is near equal gender distribution among survivors and non survivors. 

Although it has been hypothesized that mortality among patients should be higher because of the direct lung injury and has been supported by a number of studies [4, 22]. A meta-analysis [23] and Agarwal and colleagues [6] did not find any difference in mortality among patients with pulmonary ARDS versus extra pulmonary ARDS. Even in our study there is near equal distribution of pulmonary and extrapulmonary ARDS among survivors and non survivors.

In our study higher APACHE ll score representing severity of disease was significantly associated with mortality—an observation consistent with previous studies [18, 21].

It has been said that most improvement in ARDS survival observed over the past 20 years is explained by reduced case fatality in patients with a risk factor other than sepsis. Worldwide sepsis along with MOF and refractory shock has been leading cause of death in patients with ARDS [13, 18, 25]. In this study although MOF and refractory shock were significantly associated with mortality, but this was not the case with sepsis. Sepsis either as etiology or developed as an in-hospital complication though was found more in non survivors than survivors but trend did not reach statistical significance. On the contrary, in this study none of the patients developing refractory shock survived.

Interestingly refractory hypoxemia was significantly associated with mortality in this cohort and as is the case with refractory shock, none of the patients with refractory hypoxemia survived. It was present in 57.7% of non survivors—a finding inconsistent with rest of the studies. In 1985, Montgomery et al. [24] reported that refractory hypoxemia was the cause of death in less than 20% of patients with ARDS. Further studies showed similar results, [18, 25, 26] supporting the concept that ARDS is rarely, by itself, a cause of death. Although we in our institution use lung protective ventilatory strategy as the standard of care, but the reason behind this observation is unclear. This may be due to the fact that our institution lacks the facilities of extracorporeal membrane oxygenation (ECMO), inhaled nitric oxide, partial liquid ventilation, or surfactant which though failed to show consistent survival benefit in studies do show improvement in individual cases. However, it requires further clarification.

Recently, a New Berlin definition of ARDS [27] has been proposed which is endorsed by European Society of Intensive Care Medicine, the American Thoracic Society (ATS), and the Society of Critical Care Medicine (SCCM). According to it, the onset of ARDS must be acute, with bilateral lung opacities on chest X-ray and respiratory failure simply being not fully explained by cardiac failure or fluid overload. There is no need to exclude heart failure in the new ARDS definition. Three categories were developed on the basis of hypoxemia: mild (PaO_2_/FiO_2_ ≤ 300 mmHg but >200 mmHg), moderate (PaO_2_/FiO_2_ ≤ 200 mmHg but >100 mmHg), or severe (PaO_2_/FiO_2_ ≤ 100 mmHg), all on positive end expiratory pressure (PEEP) of 5+. These groups, according to consensus panel, were associated with increased mortality (27%, 32%, and 45%, resp.). As we recruited patients on the basis of older definition so we did not categorize them into mild, moderate, and severe groups and also did not include the ventilatory parameters, which definitely is a limitation to our study in view of current definition.

Apart from the above mentioned, there were certain other limitations of our study. It was a single centre study. Sample size was small and because of small sample size only univariate analysis was possible, and we cannot calculate the independent risk factor of mortality using multivariate analysis.

## 6. Conclusion

ARDS is a syndrome associated with high mortality among ICU patients. In this study the mortality rate was 56%. Age, gender, and etiology of ARDS and sepsis were equally distributed among survivors and nonsurvivors. High APACHE score, multiorgan failure, and refractory shock were associated with high mortality as in previous studies. On the contrary to most of the other recent studies, refractory hypoxemia was found to be a leading cause of death in this group of patients.

Because of small sample size these results are not generalizable, but yet to our knowledge this is the first study in Pakistan on this subject. Further studies are required preferably multicentre and with large sample size to make these results meaningful.

## Figures and Tables

**Table 1:** Univariate analysis of risk factors of mortality.

Characters	Survivors	Non survivors	*P*-value
Age			
>65 Y	10% (*N* = 2)	34.6% (*N* = 9)	0.08
<65 Y	90% (*N* = 18)	65.4% (*N* = 17)	
Gender			
Male	45% (*N* = 9)	65.4% (*N* = 17)	0.16
Female	55% (*N* = 11)	34.6% (*N* = 9)	
APACHE			
>20	25% (*N* = 5)	69.2% (*N* = 18)	0.003
<20	75% (*N* = 15)	30.8% (*N* = 8)	
Sepsis	35% (*N* = 7)	61.5% (*N* = 16)	0.07
MOF (Multi organ failure)	35% (*N* = 7)	92.3% (*N* = 24)	<0.001
Refractory shock	0	53.8% (*N* = 14)	<0.001
Refractory hypoxemia	0	57.7% (*N* = 15)	<0.001
